# Supplementary material for: Differential vasoproliferative traits of Bartonella henselae strains associated with autotransporter BafA variants
Source: Microbiol Spectr. 2024 Nov 29;13(1):e01925-24. doi: 10.1128/spectrum.01925-24 (PMC11705867; doi:10.1128/spectrum.01925-24)
Supplement: Supplemental figures — Fig. S1 to S5. [file spectrum.01925-24-s0001.pdf]

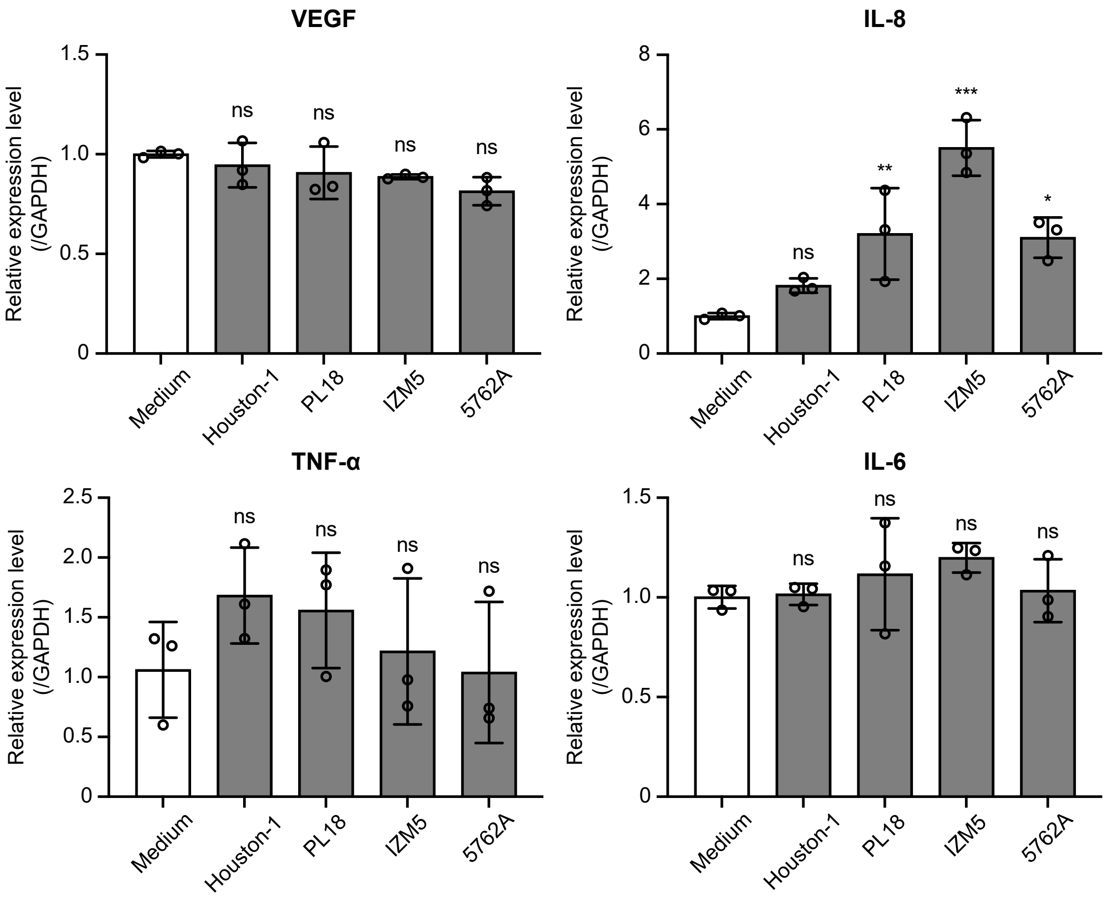

Figure S1. Cytokine gene expression in HUVECs after *B. henselae* infection. Relative mRNA levels of VEGF, IL-8, IL-6, and TNF-α in HUVECs 3 hours post-infection (MOI 1,000), measured by qRT-PCR. Results are shown as fold changes from uninfected controls. Bars: means ± SDs (n = 3; open circles). A one-way ANOVA with Dunnett’ s test: ns, not significant; *, $P < 0.05$; **, $P < 0.01$; ***, $P < 0.001$. Notations above bars indicate comparisons to the control group.

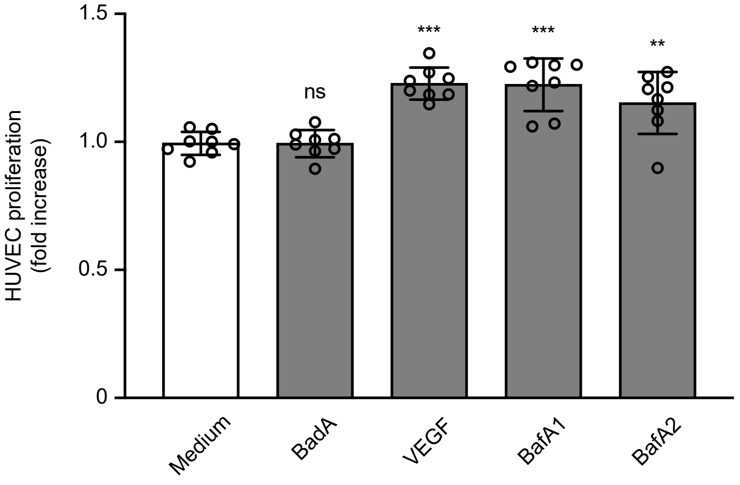

Figure S2. HUVEC proliferation in response to recombinant proteins and VEGF. HUVECs treated with the BadA stalk fragment, or BafA1, or BafA2, or VEGF (300 ng/mL each) for 2 days. Results are shown as fold changes from untreated controls. Bars: means ± SDs (*n* = 8; open circles). A one-way ANOVA with Dunnett's test: ns, not significant; **, $P < 0.01$; ***, $P < 0.001$. Notations above bars indicate comparisons to the control group.

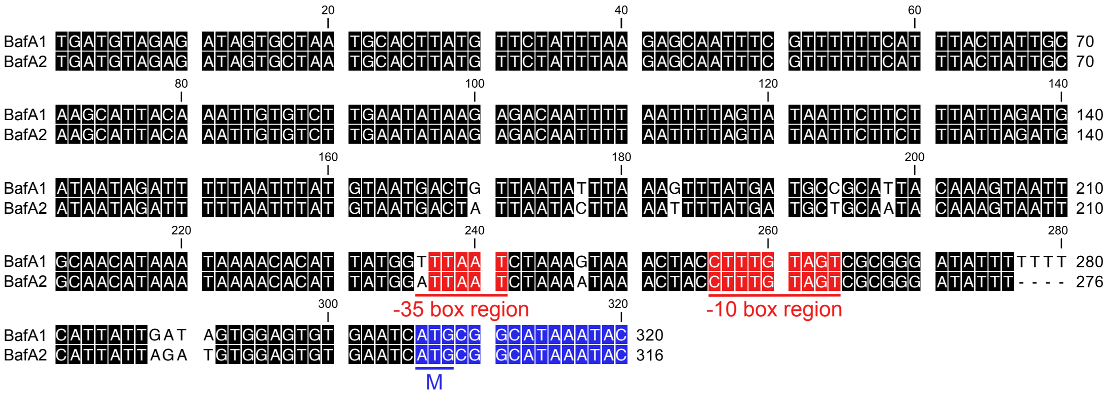

Figure S3. Putative promoter regions of the *bafA* gene. Sequence alignment upstream of the *bafA* transcription initiation site. Red underlines: predicted -10 and -35 box regions by the BPROM program (Softberry, Inc.). Blue underline: start codon (methionine, M).

A

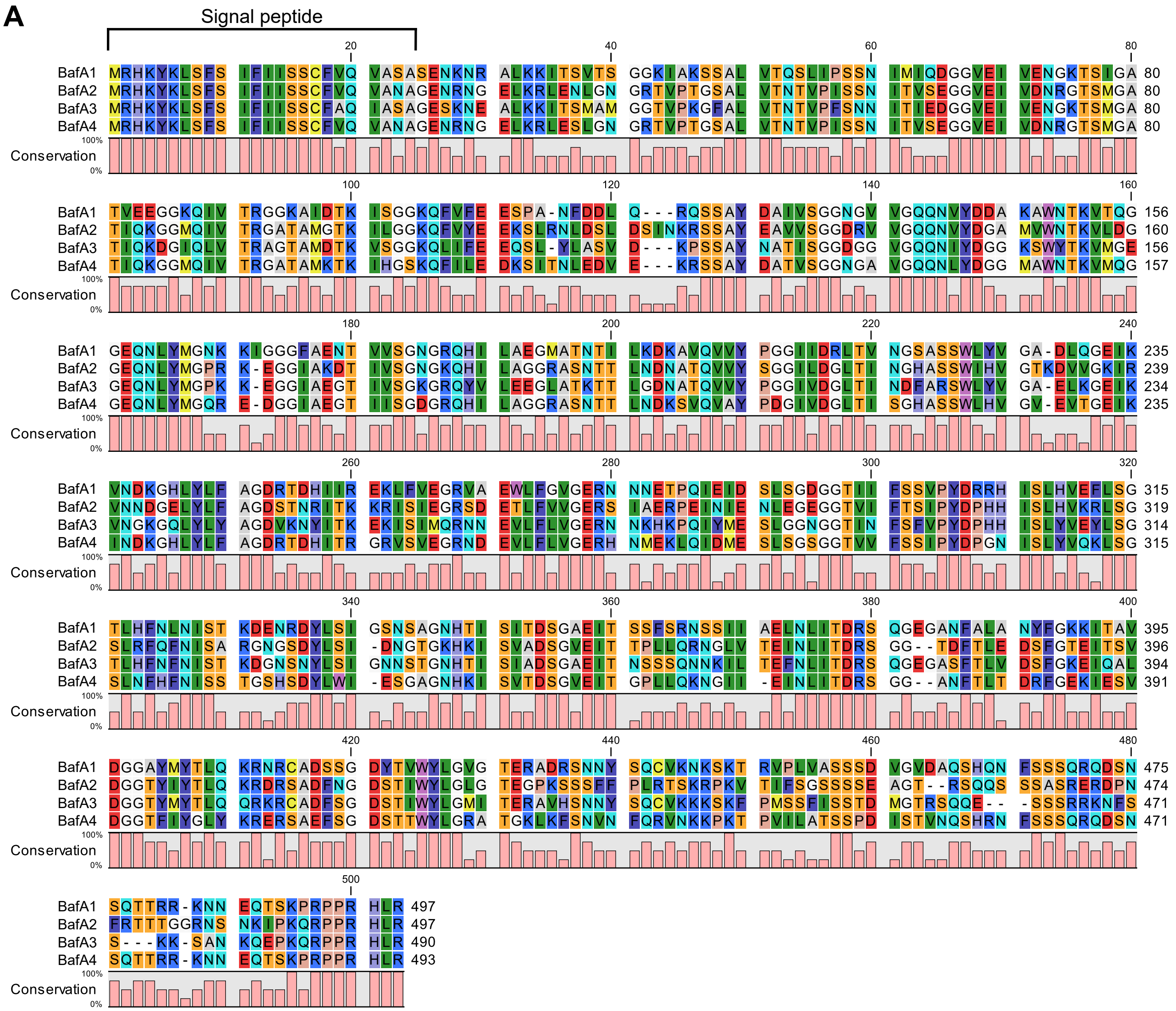

B

	BafA2	BafA3	BafA4
BafA1	53.2%	62.1%	60.3%
BafA2		56.4%	66.1%
BafA3			57.2%
BafA4			

Figure S4. Comparison of BafA variant passenger domains. (A) Amino acid sequence alignment of BafA1-4 passenger domains. (B) Percentage identity matrix of pairwise sequence comparisons.

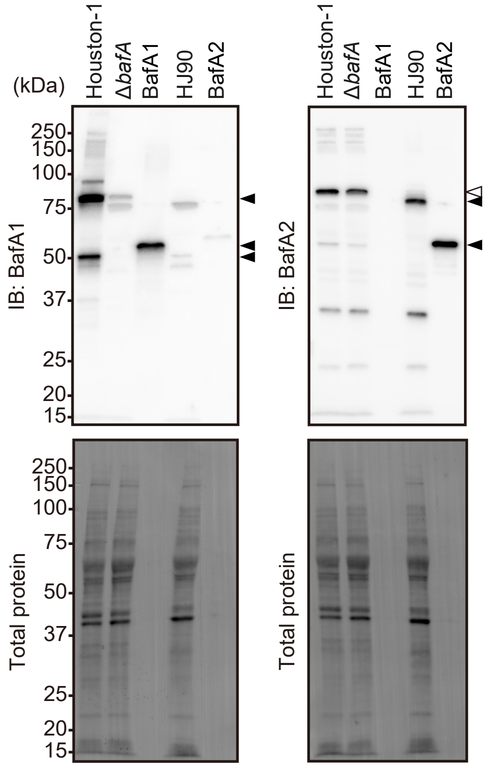

Figure S5. Antibody specificity for BafA1 and BafA2. Western blot of BafA from *B. henselae*-HUVEC cocultures (2 days) with variant-specific antibodies (top) and total proteins (bottom). Houston-1: BafA1 strain; HJ90: BafA2 strain. Controls: recombinant BafA (2 ng/lane, positive) and *ΔbafA* lysate (negative). Black arrowheads: specific bands; white: non-specific. Anti-BafA1 detects intact BafA (~90 kDa) and the passenger domain (~50 kDa) in Houston-1. Anti-BafA2 detects intact BafA (~90 kDa) in HJ90 with some non-specific bands. White arrowhead bands in both Houston-1 and *ΔbafA* are deemed non-specific.
