# Supplementary material for: Differential vasoproliferative traits of Bartonella henselae strains associated with autotransporter BafA variants
Source: Microbiol Spectr. 2024 Nov 29;13(1):e01925-24. doi: 10.1128/spectrum.01925-24 (PMC11705867; doi:10.1128/spectrum.01925-24)
Supplement: Supplemental material — Supplemental methods. [file spectrum.01925-24-s0002.docx]

**Text S1: Supplemental Methods**

**RNA extraction and quantitative RT‒PCR.** HUVECs were infected with *B. henselae* strains (MOI 1,000) and cultured at 37℃ for 3 h in 5% CO_2_. Total RNA was extracted using the RNeasy Mini Kit (Qiagen) and 500 ng was reverse transcribed with the QuantiTect Reverse Transcription Kit (Qiagen). Quantitative RT‒PCR was performed using THUNDERBIRD Next SYBR qPCR Mix (Toyobo, Japan) with the primers listed in Table S1 (1, 2). GAPDH served as an endogenous control. Relative gene expression was calculated using the 2^(− ΔΔCT) method.

**HUVEC proliferation assay.** HUVECs (7,000 cells/well) were seeded in 96-well plates with EGM-2. After an incubation and medium exchange, cells were treated with 300 ng of the recombinant BadA stalk, BafA1 or BafA2, or VEGF-A_165_ (PeproTech, Cranbury, NJ) for 2 days. HUVEC proliferation was evaluated as described in the main text.

**Evaluation of antibody specificity.** *B. henselae* strains (Houston-1, *ΔbafA* [strain 623-125 (3)], and HJ90; MOI of 1,000) were cocultured with HUVECs for 2 days. Cells were lysed with 50 μL BugBuster Master Mix per well. Lysates were mixed with 10 μL 6× Sample Buffer Solution containing Reducing Reagent (Nacalai Tesque). SDS-PAGE and western blotting using anti-BafA1 and anti-BafA2 antibodies were performed as described in the main text. Recombinant BafAs (2 ng) served as controls.

**References**

1. Hu L, Zang MD, Wang HX, Li JF, Su LP, Yan M, Li C, Yang QM, Liu BY, Zhu ZG. 2016. Biglycan stimulates VEGF expression in endothelial cells by activating the TLR signaling pathway. ***Mol Oncol*** 10:1473-1484.

2. Gao H, Zhang Q, Chen J, Cooper DKC, Hara H, Chen P, Wei L, Zhao Y, Xu J, Li Z, Cai Z, Luan S, Mou L. 2018. Porcine IL-6, IL-1beta, and TNF-alpha regulate the expression of pro-inflammatory-related genes and tissue factor in human umbilical vein endothelial cells. ***Xenotransplantation*** 25:e12408.

3. Tsukamoto K, Shinzawa N, Kawai A, Suzuki M, Kidoya H, Takakura N, Yamaguchi H, Kameyama T, Inagaki H, Kurahashi H, Horiguchi Y, Doi Y. 2020. The Bartonella autotransporter BafA activates the host VEGF pathway to drive angiogenesis. ***Nat Commun*** 11:3571.
