# Supplementary material for: Differential vasoproliferative traits of Bartonella henselae strains associated with autotransporter BafA variants
Source: Microbiol Spectr. 2024 Nov 29;13(1):e01925-24. doi: 10.1128/spectrum.01925-24 (PMC11705867; doi:10.1128/spectrum.01925-24)
Supplement: Supplemental tables — Tables S1 and S2. [file spectrum.01925-24-s0003.docx]

**Table S1. Primers used in the present study**

| **Primers** | **Sequences (5’-3’)** | **Target** | **Reference** |
| --- | --- | --- | --- |
| BafA-305-Fw | CTGATGTAGAGATAGTGCTA | BafA | This study |
| BafA-93-Rv | CTCTCACAGAAAACTCTCTC |  |  |
| BafA1-Seq-Fw | GTGGTTTCAGGGAATGGTAG |  |  |
| BafA2-Seq-Fw | AAGTTTTAGACGGGGGAGAG |  |  |
| BafA12-Seq-Rv | ACACCACTGACACCTAAAAC |  |  |
| NheI-BadA-Fw | CAGCCATATGGCTAGCAAAGCATTAAGGGGAATGATATCAG | BadA stalk region | Riess *et al.* (2004) |
| NheI-BadA-Rv | CTGTCCACCAGTCATGCTAGCTCAAGTACGCTTATCACTTTTGTTATTAGC |  |  |
| NheI-Bh-Fw | TCTCGCTAGCAGTGAGAATAAGAATAGGGCG | BafA1 | Tsukamoto *et al.* (2020) |
| SalI-Bh513-Rv | GACTGTCGACTCATTTTTCTAAAAATGGAATCGTC |  |  |
| NheI-BAF2-Fw | CAGCCATATGGCTAGCGGCGAGAACAGGAATGGAGAG | BafA2 | This study |
| NheI-BAF2-Rv | CTGTCCACCAGTCATGCTAGCTCATCTTAAATGCCGTGGAGGTCTTTG |  |  |
| RT-GAPDH-Fw | GGACCTGACCTGCCGTCTAG | GAPDH | Hu *et al.* (2016) |
| RT-GAPDH-Rv | GTAGCCCAGGATGCCCTTGA |  |  |
| RT-VEGF-Fw | AGGGCAGAATCATCACGAAGT | VEGF-A |  |
| RT-VEGF-Rv | AGGGTCTCGATTGGATGGCA |  |  |
| RT-IL6-Fw | ACTCACCTCTTCAGAACGAATTG | IL-6 | Gao *et al.* (2018) |
| RT-IL6-Rv | CCATCTTTGGAAGGTTCAGGTTG |  |  |
| RT-IL8-Fw | ACTGAGAGTGATTGAGAGTGGAC | IL-8 |  |
| RT-IL8-Rv | AACCCTCTGCACCCAGTTTTC |  |  |
| RT-TNFα-Fw | GAGGCCAAGCCCTGGTATG | TNF-α |  |
| RT-TNFα-Rv | CGGGCCGATTGATCTCAGC |  |  |

**Table S2. NCBI accession numbers of sequences used in the analysis in Fig. 5**

| **Strain** | **NCBI Accession number** | |
| --- | --- | --- |
|  | **Genome assembly** | **BafA** |
| **Clade 1 strains** | | |
| Houston-1 (ATCC 49882) | GCA_000046705 | WP_011180481 |
| JK 51 | GCA_000516675 | WP_011180481 |
| JK 50 | GCA_000516695 | WP_011180481 |
| FDAARGOS_175 | GCA_001525625 | WP_011180481 |
| FDAARGOS_1462 | GCA_019930925 | WP_011180481 |
| 88-64 Oklahoma | GCA_021560425 | WP_011180481 |
| G-5436 | GCA_021560445 | WP_011180481 |
| Berlin-I | GCA_021560465 | WP_011180481 |
| BH13 | GCA_030385955 | WP_011180481 |
| BM1374163 | GCA_000612965 | WP_038525149 |
| BM1374164 | GCA_902728145 | WP_038525149 |
| MVT02 | GCA_001291465 | WP_038525149 |
| **Clade 2 strains** | | |
| JK 41 | GCA_000516735 | WP_034454260 |
| Zeus | GCA_000708485 | WP_034454260 |
| JK 53 | GCA_000708545 | WP_034454260 |
| FR96/BK38 | GCA_021560405 | WP_034454260 |
| BH52 | GCA_030385885 | WP_034454260 |
| BH54 | GCA_030385975 | WP_034454260 |
| BH16 | GCA_030386015 | WP_034454260 |
| BH63 | GCA_030386055 | WP_034454260 |
| BH56 | GCA_030386075 | WP_034454260 |
| BH40 | GCA_030386135 | WP_034454260 |
| BH25 | GCA_030386175 | WP_034454260 |
| BH20 | GCA_030386185 | WP_034454260 |
| BH1 | GCA_030385935 | WP_289818562 |
| BH27 | GCA_030386095 | WP_289818562 |
| BH38 | GCA_030386215 | WP_289818562 |
| BH58 | GCA_030385965 | WP_289818562 |
| BH61 | GCA_030386025 | WP_289818562 |
| **Clade 3 strains** | | |
| BH45 | GCA_030386155 | WP_082250487 |
| A112 | GCA_001932145 | WP_082250487 |
| A233 | GCA_001932165 | WP_082250487 |
| A121 | GCA_001932175 | WP_082250487 |
| U4 | GCA_001932295 | WP_082250487 |
| Marseille/ URLLY-8 | GCA_021560525 | WP_082250487 |
| BM1374165 | GCA_000612765 | WP_038487267 |
| **Clade 4 strains** | | |
| A244 | GCA_001932235 | WP_082251757 |
| FR96/BK3 | GCA_021560385 | WP_082251757 |
